# Advancing Point-of-Care (PoC) Testing Using Human Saliva as Liquid Biopsy

**DOI:** 10.3390/diagnostics7030039

**Published:** 2017-07-04

**Authors:** Rabia Sannam Khan, Zohaib Khurshid, Faris Yahya Ibrahim Asiri

**Affiliations:** 1 Department of Oral Pathology, College of Dentistry, Baqai University, Super Highway, P.O.Box: 2407, Karachi 74600, Pakistan; rabia.sannam.khan@gmail.com; 2 Prosthodontics and Implantology, College of Dentistry, King Faisal University, Al-Ahsa 31982, Saudi Arabia; 3 Department of Preventive Dentistry, College of Dentistry, King Faisal University, Al-Ahsa 31982, Saudi Arabia; fasiri@kfu.edu.sa

**Keywords:** saliva, diagnostic toolboxes, biomarkers, the point of care, diseases

## Abstract

Salivary diagnostics is an emerging field for the encroachment of point of care technology (PoCT). The necessity of the development of point-of-care (PoC) technology, the potential of saliva, identification and validation of biomarkers through salivary diagnostic toolboxes, and a broad overview of emerging technologies is discussed in this review. Furthermore, novel advanced techniques incorporated in devices for the early detection and diagnosis of several oral and systemic diseases in a non-invasive, easily-monitored, less time consuming, and in a personalised way is explicated. The latest technology detection systems and clinical utilities of saliva as a liquid biopsy, electric field-induced release and measurement (EFIRM), biosensors, smartphone technology, microfluidics, paper-based technology, and how their futuristic perspectives can improve salivary diagnostics and reduce hospital stays by replacing it with chairside screening is also highlighted.

## 1. Introduction

Laboratory testing remains the dominant mainstay for analytical processes of a large number of samples involving the disciplines of biochemistry, haematology, microbiology, anatomical pathology, and much more [1]. Due to the limitations and pressure on healthcare budgets faced by a very large number of countries, primary care is best suited for the world to reduce expenses instead of secondary and tertiary hospitals. Poverty, chronic disease, infections lead to significant problems in developing the world, and adequate diagnostic testing turns out to be difficult to meet the needs. Hence, consequently, initiatives in making solid models using point-of-care technology (PoCT) came into existence [2].

### 1.1. Paradigm Shift from Central Laboratory (CL) to Point-of-Care

A self-monitoring blood glucose meter, coagulation (INR), and pregnancy testing kits using urine samples are well-known examples of PoCT and has become over-the-counter products to be sold in the market. Saliva is predicted to be a substitute for blood, collected non-invasively for the diagnosis of oral and systemic diseases. Thus, PoCT replaces the specialist testing centres by using the samples other than blood and urine [3]. For the development of PoCT devices, minimum risk of infection with no mental and physical pain is of utmost importance to consider, in addition to automation, integration, multiplexed detection ability, quick analysis, small sample size, and minimal training as the primary goals of modern medicine [4]. With the advent of the struggle in the growing potential of developing PoCT, the World Health Organization (WHO) provided guidelines which had the features for designing devices, known as the ASSURED criteria, which indicated that devices had to be affordable, sensitive, specific, user-friendly, rapid and robust, with no complex equipment, and be delivered to end users efficiently.

The precision to central lab testing is being facilitated by the use of biomarkers and saliva is a viable biofluid for diagnostic applications. The human saliva revolution in medical and dental sciences through its property as a “*mirror of body health*” in the last decade brought many disease detections through its compositional changes in disease conditions [5]. Human oral cavity consists of different sources, such as salivary glands (major and minor), gingival crevicular fluid (GCF), microbes, and oral epithelial sheds for the production of whole mouth fluid (WMF) [6,7,8,9,10,11,12]. This fluid is 99% water, but 1% consists of DNA, mRNA, microRNA, proteins, metabolites, and microbiota which are utilised as a diagnostic fluid for disease analysis and even for forensic analysis [13,14]. In this particular review, we present the current knowledge of saliva, diagnostic toolboxes, how PoC technology works, developed PoC devices for detection of various diseases, and the future prospects of utilising saliva as a diagnostic tool.

### 1.2. Saliva in the Diagnosis of Oral and Systemic Diseases

**Figure 1:**
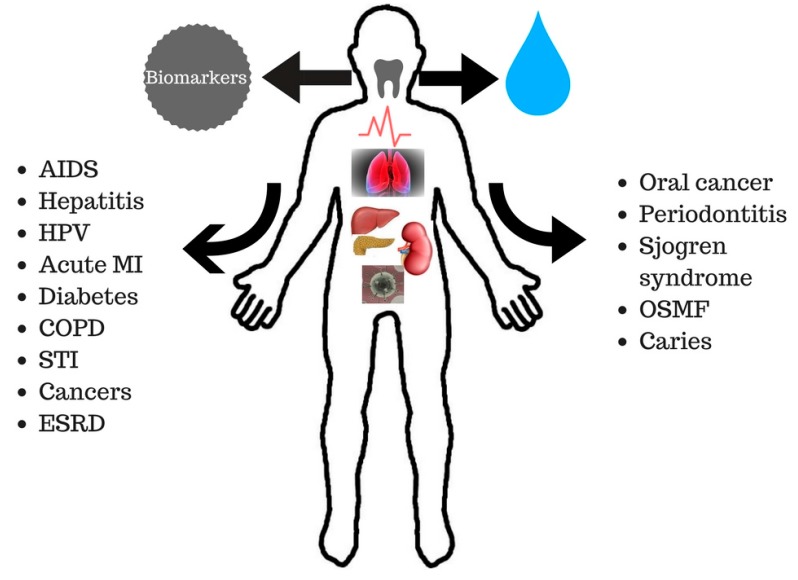
Depiction of the detection of various oral and systemic diseases through salivary biomarkers. Acquired Immuno Defeciency Syndrome (AIDS), Human Papilloma Virus (HPV), Myocardial infarction (MI), Chronic Obstructive Pulmonary Disease (COPD), Sexually Transmitted Infection (STI), End Stage Renal Failure (ESRD) and Oral Squamous Muccous Fibrosis (OSMF).

Saliva is a complex fluid containing various enzymes, electrolytes, proteins, nucleic acids, antimicrobial constituents, hormones, cytokines, and antibodies. Its composition virtually reflects the entire state of health and disease in a body and it has the potential of being a diagnostic medium for a broad range of diseases [15,16], such as in the detection of periodontal diseases, caries risk assessments, breast cancer, oral cancers, salivary gland diseases, HIV, and much more [16]. However, the effects of alteration in the salivary composition are seen in the lipid profile of cystic fibrosis patients, which is markedly changed in comparison to healthy subjects [17]. The submandibular gland saliva of cystic fibrosis patients contains 66% more lipids per 100 mL of saliva than that of a healthy subject. The salivary fatty acid profile can be a good indicator for the early detection of tumorigenesis processes and cardiovascular diseases. The increase in production of salivary arachidonic acid, relevant for their eicosanoid production related to the tumorigenesis process and cardiovascular diseases, is influenced by dietary fat intake [18]. Figure 1 portrays alterations of biomarkers in various body organs in diseased states through which saliva can detect a variety of oral and systemic diseases.

Similarly, in Sjogren’s syndrome (SS) the saliva flow is compromised in the patient due to ductal changes by lymphocytic infiltration and fibrosis of the salivary glands and the patient also suffers from the consequences of dental caries, infections dysphagia, and other oral pain [19]. The lipid profile of SS patients is twice higher than an average healthy person and elevated level of cytokines (IgA, IgG, interleukin-6, and prostaglandins-E2) and antibodies. Many salivary proteins were reported as biomarkers for SS diagnosis; for example, profilin [20], anhydrase-I [21], IL-4, IL-5 [22], MxA [23], and CXCL13 [24,25]. Human saliva has a proven diagnostic role in the detection of cardiovascular diseases, reported in studies examining whole mouth salivary biomarkers e.g., C-reactive proteins (CRP) [26], cardiac troponin (cTn) [27], creatine phosphokinase [28], and NT-ProBNP [29]. Diabetes is another common disease faced all around the world and developing rapidly due to dietary habits, genetic, and other systematic disease-related complications [30]. Due to its non-invasiveness, a cheap and easy sampling of saliva is attractive as a diagnostic fluid for diabetes analysis [31]. Previously reported studies concluded the different biomarkers for diabetes detection in early stages. A recently-reported study saw a marked alteration in levels of salivary glucose, amylase, calcium, and phosphorus in comparison to serum from diabetic and non-diabetic patients [32], as seen in Table 1 below.

The current review presents a broad overview on salivary diagnostics, the validation of biomarkers (diagnostic targets) through diagnostic toolboxes, discussion related to new biomarker-related PoC platforms, the latest emerging PoC technologies including highlights on biosensors, biological micro-electro-mechanical systems (BioMEMS), microfluidics/paper based technology, electric field-induced release and measurement (EFIRM), and smartphone-based biosensors their functions and clinical utility in medical field.

## 2. Point-of-Care Technology: An Overview

Point-of-care (PoC) technology in diagnostics tends to evaluate biomarkers that are suggestive of underlying biological or physical characteristics of an individual. Therefore, biomarkers determine the risk and severity of the disease, as well as the individual’s response to treatment [15]. In addition to it, diagnostic methodologies other than biomarkers also include biochip and biosensor systems. Biochip systems obtain the requisite volume of saliva for testing whereas the biosensor system is the analytical high-sensitive technology for the detection of biomarkers [50]. Accurate PoC diagnostics needs no pre-processing and screening for biomarker identification on top of it non-invasive testing, as seen in already-patented devices, such as for the detection of oral cancer known as the Oral Fluid NanoSensor Test (OFNASET), the detection of human papillomavirus (HPV) infection OraRisk HPV test, and the diagnosis of periodontal diseases PerioPath [51,52]. Moreover, measuring the molecular level biomarkers in the form of proteins, mRNA, DNA, electrolytes, and small molecules requires techniques of microfabrication, such as in the developed micro/nanoelectromechanical systems (MEMS/NEMS) [53], and current emerging technologies provide new avenues of PoC diagnostics in the variety of “lab-on-chip” techniques which integrates the complexities of lab procedures on a computer chip in the size of a device that, hence, gives an opportunity to detect and diagnose multiple diseases simultaneously with the help of biomarkers [54].

### 2.1. Diagnostic Targets

Salivary diagnostics needs appropriate identification and validation of biomarkers for the detection of diseases, and a biomarker is a quantifiable parameter that can interact physiologically and biochemically at a molecular or cellular level, which sequentially acts as an indicator of normal, pathological, and interventional behaviours of the body’s response [55,56]. Biomarkers include several classes, such as proteins, DNA, RNA, metabolites, and microbes, so collectively these all are used for diagnosis of several diseases and are called a molecular signature [57].

### 2.2. Diagnostic Toolboxes

Major salivary diagnostic tool boxes include proteomes, metabolomes, genomes (transcriptome, epigenome), microbiomes, and immunologic categories. Table 2 shows the variety of methods used to analyse molecules for the investigation and validation of biomarkers. Proteomes make up the biological system and could be employed for the detection of diabetes, periodontitis, caries, cystic fibrosis, AIDS, OSCC, breast cancer, lung cancer, pancreatic cancer, Sjogren’s syndrome, and many more through mass spectroscopy, 2D gel electrophoresis, ELISA and protein immunoblot techniques [58,59,60,61]. However, mRNA and DNA come under salivary transcriptomes and genomes, and their profiling through gene chip arrays, DNA hybridization, qPCR, and gel electrophoresis helps in the detection of OSCC, as conducted by Li et al. [62], and in Sjogren’s syndrome, hepatitis, HIV, etc. The metabolic investigation, on the other hand, requires and uses gas chromatography mass spectrometry, nuclear magnetic resonance spectroscopy, and high-performance liquid chromatography [63] for the detection of diabetes, lung, pancreatic, breast cancers, and Sjogren’s syndrome [64,65]. In the same way, the salivary microbiome and immnomics used different methods, as mentioned in the table for the detection of infectious diseases, HIV, hepatitis, malaria, dengue, Ebola virus, cytomegalovirus, herpes infection, and countless other diseases [66,67,68].

### 2.3. Salivary Biomarker-Based PoC Platforms

In Table 3, shows analysis system based on different technologies applied on saliva for the detection of biomarkers to reduce the time duration and early diagnosis of certain diseases. As shown in table it is revealed that single and multiplexed systems were being used such as MEMS, ORI, chromatography test strips and several salivary diagnostics devices (USA) for the detection of proteins solely, also proteins and nucleic acids altogether (for e.g., IL-8, MMP-8, a-amylase, HIV, HCV) with lowering the time limit as much little as one minute. These technologies, therefore, reduces the invasive procedures significantly to a larger level [16].

## 3. Emerging Novel PoC Technologies

### 3.1. Biosensors

The biosensor is a bioanalytical device which has the ability to mimic any biological material, i.e., antibodies/antigens, nucleic acids, cellular structures or enzymes. That organic material is integrated into a transducing microsystem. The transducer could be electrochemical, thermometric, optical, piezoelectric, or magnetic, and this is called label-free detection [74], while label-based detection includes fluorescent immunoassays, FRET, and quantum dots. Biosensors function with the biorecognition of particular elements for particular targeted analytes and the maintenance of selectivity and sensitivity in the existence of other interfering compounds [75]. In the medical field, biosensor applications are growing rapidly, such as for the diagnosis of diabetes mellitus, urinary tract infections (UTI), identification of end-stage heart failure, and acute leukaemias (Table 1) [76].

### 3.2. Fluorescent Biosensors

Fluorescent biosensors can be used for cancer, drug discovery, arthritis, cardiovascular and neurodegenerative diseases, viral infections, chronic myeloid leukemia, and many more, by using the principal of high throughput screening approaches, the use of fluorescent probes in gene expression, localization of protein in cell cycle, apoptosis, signal transduction, and transcription. For the detection of chronic myeloid leukaemia, a genetically-encoded FRET biosensor was developed to detect Bcr-Abl kinase activity to see the correlation, it was further used to check the response to treatment, drug resistance, and predictive values for alternative therapeutics [77]. Moreover, Lee et al. applied hafnium oxide in a novel biosensor for the detection of human interleukin (IL-10) (atomic layer deposited hafnium oxide gate dielectrics for charge-based biosensors) [78]. Applications of nanomaterial biosensors give opportunities for a new generation of biosensor technologies that can be broadly used in monitoring, diagnosis, control, and analysis.

### 3.3. Biological Micro-Electro-Mechanical Systems (BioMEMS)

PoC lab-on-chip systems use small and simply-constructed (BioMEMS) devices for the detection of biological and chemical agents. BioMEMS are utilized for the detection of cells, proteins, microorganisms, viruses, and DNA in biological samples. They are based on micro/nanoscale fabrication systems which help in increasing the sensitivity of results from sensors, increased reliability, increased performance, reduced detection time, and cost effectiveness. It has label-free detection techniques, including micro-cantilevers, surface plasmon resonance (SPR), quartz crystal microbalances (QCM), and organic field-effect transistors (BioFETs) [79]. BioMEMS are used for a range of applications, such as for drug delivery, cardio MEMS to monitor heart patients, hearing aids, insulin micropumps, endoscopic pills, and retinal prosthesis [80,81] (Table 3). By blending molecular biology with computational systems, the major nanotechnology achievement could be bio-nano-electro-mechanical systems (BioNEMS) in the future for further improvements in the medical sector.

### 3.4. Microfluidics/Paper-Based Technology

Microfluidic applications operate on integrated microfabrication and specific physiochemical properties. Initially, the use of silicon, inorganic glass, and ceramic was used in microfluidic devices which have been vastly replaced by the soft and rigid thermostatic and thermoplastic materials and, finally, into paper-based technologies, using biodegradable and hydrogel materials [82]. At Harvard University in 2007 microfluidic paper-based analytical devices (μPADs) were pioneered by Whitesides. Paper is porous and hydrophilic, therefore, it provides a platform for the fabrication of microfluidic channels by patterning the paper with 2D and 3D μPADs and having a variety of assay designs. They are used in the detection of urine metabolites, blood glucose, pH value, liver function, and infectious agents, and they are also widely used in pregnancy test kits [83].

### 3.5. Electric Field-Induced Release and Measurement (EFIRM)

A liquid biopsy technique called EFIRM, uses readout enzymes and immobilised probes for the capturing and detection of biomarkers from biofluids. EFIRM uses the electrochemical method to facilitate nucleic acid hybridization. This approach is advantageous for accurate detection of RNA, protein biomarker targets in exosomes. Additionally, without the extraction of DNA and nucleic acid, EFIRM can analyse the mutation status within an hour. IL-8 protein and IL-8 mRNA markers for oral cancer, non-squamous cell lung cancer (NSCLC) oncogenic mutation, and epidermal growth factor receptor (EGFR) mutation in non-small cell lung cancer can be detected through this EFIRM system [57,84].

### 3.6. Smartphone-Based Biosensors

**Figure 2:**
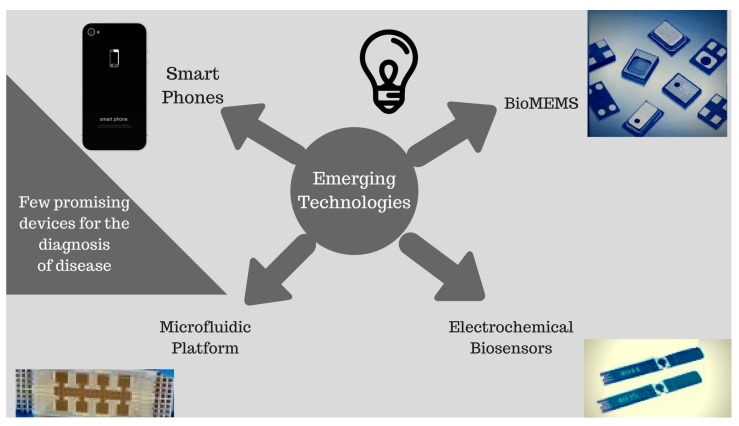
Illustration of a range of promising emerging methods of PoC technologies.

Smartphones operate similarly to miniature computers, acting as cheap, portable analytical laboratory devices. They are helpful for the detection, and diagnosis, of various diseases, such as cancer, tuberculosis, and the self-monitoring of blood glucose. Figure 2 illustrates the few shapes and designs of currently-used PoC technologies while Table 4 shows all the functions, techniques, and clinical utilities of these techniques in a concise manner.

Recent advancements in smartphones electronics and new app development have made its use as a smart detector by having all the mixed optical methods, i.e., fluorescence, surface plasmon resonance (SPR), reflectance, absorbance, bio-chemiluminescence, and electrochemiluminescence [85]. Amongst them all SPR gained importance because of its high sensitivity, label-free, microfluidic technique. Conventional SPR utilizes planar thin gold film while localized SPR (LSPR) contains metal nanostructures [86]. Hence, SPR becomes the powerful tool in biomedical application such as for the study of several DNA, RNA, proteins, lipids, carbohydrates, even mutation detection [87]. Moreover, Lee et al. described the smartphone’s function as a compact microscope in which ambient illumination as a light source was being used instead of a chip-scale method. This lensless imaging scheme allows sub-micron resolution and the built-in android application brings simplicity, robustness, and employability for several field applications [88].

## 4. Future Direction and Conclusions

This article gives an overview of research in molecular diagnostics, microbiology, and immunology. Routine laboratory testing includes the majority of haematology testing, clinical chemistry, and immunochemistry by using high-throughput instrumentation. Therefore, salivary PoCT diagnostics is replacing the central laboratory and offers efficient, fast, quick and easy automation. Since the emphasis is switching more towards prevention and early detection of a variety of diseases, development of small wireless devices has made a dramatic impact on healthcare services. The next decade will bring breakthroughs in terms of precision, efficiency, and bedside monitoring instead of hospital setups.

Personalised medicine, with the help of biosensors, lab-on-chip systems, individual genetics, smartphones monitoring parameters, and microfluidic devices, will improve the primary healthcare system. Moreover, it allows clinicians to be accurate, to be more consistent, to capture clinical data quickly, provide patient satisfaction, and streamline workflow. Salivary diagnostics’ impact on the healthcare system is enormous, being non-invasive, convenient, and well-credentialed, while bioinformatics introduction will make standards and performance higher and improved.

## Figures and Tables

**Table 1:** Description of Point-of-care (PoC) devices for detection of diseases through specific salivary biomarkers.

Salivary Biomarkers	Diseases/Conditions	Developed PoC	References
A-amylase	Clinical judgment for stress-induced disease	Salivary α-Amylase (sAA) biosensor system	[33]
HIV	AIDS	Oraquick, tablet-based kiosks	[34,35]
Hep C	Hepatitis	OraQuick	[36]
HPV	HPV-associated cancers, sexually transmitted diseases	simple fluorescent and colorimetric assay that enables DNA and RNA detection	[37]
Cortisol	Stress levels	Label-free chemiresistor immuno-sensor	[38]
Proteins (Dipeptidyl peptidase etc.), metabolites, DNA	Periodontitis	Integrated Microfluidic Platform for Oral Diagnostics (IMPOD), lab-on-a-chip (LOC)	[39]
C-reactive protein, myoglobin, and myeloperoxidase	Acute Myocardial Infarction	Luminex, lab-on-a-chip methods	[40]
Cytokines	Asthma and chronic obstructive pulmonary disease (COPD)	Fiber-optic microsphere-based antibody array	[41]
IL-8, IL-8mRNA	Oral Cancer	Electrochemical magneto biosensors	[42]
(NO_2_− and uric acid), and pulmonary inflammation biomarkers	End-stage renal disease (ESRD), asthma and chronic obstructive pulmonary disease (COPD) patients	Optical fibre microarrays	[43]
Salivary nicotine metabolites	Smoking/tobacco use	Point of care test for salivary nicotine metabolites	[44]
*Porphyromonas gingivalis*	chronic periodontitis	*P. gingivalis* saliva kit	
Gonorrhoea and chlamydia	Sexually transmitted infections (STIs)	Oral STI point-of-care (PoC)	[36]
Salivary anti-Ro60 and anti-Ro52 Antibody Profiles	Sjögren’s Syndrome	Luciferase Immunoprecipitation Systems (LIPS)	[45]
Salivary glucose	Diabetes	Glucose monitoring using saliva nanostructured biosensor	[46,47]
cRP, MPo, ctnl, Myo, cK-MB, d-dimer, apoa1, apoB, BnP, nt-proBnP, scd40l, McP-1, adiponectin	Cardiovascular disease (CVD)	Programmable bio-nanochip (P-BNC) system	[48]
cea, ca125, Her2-neu, Psa (free and complexed)	Cancer	Programmable bio-nanochip (P-BNC) system, 2D nanomaterials	[48,49]

**Table 2:** Methods used for the evaluation of diagnostic toolboxes.

Diagnostic Toolbox	Methods of Evaluation	Molecules to Be Analysed	References
Proteomics	Mass spectroscopy and 2D gel electrophoresis, ELISA, protein immunoblot techniques	Post-translational modifications and protein-enzyme complexes	[69]
Genomics (Transcriptomics and Epigenomics)	Gene chip arrays, DNA hybridization, qPCR, and gel electrophoresis	DNA, RNA and mRNA	[70]
Metabolomics	Nuclear magnetic resonance spectroscopy (NMR), gas chromatography-mass spectrometry, direct flow injection/liquid chromatography-mass spectrometry, inductively coupled plasma mass spectrometry, and high-performance liquid chromatography (HPLC), capillary electrophoresis time of flight mass spectroscopy	Small molecules end products of metabolic processes in the body such as organic species, together with non-protein hormones (epinephrine, peptide hormones and cortisol).	[71]
Microbiome	Bacterial microarrays, DNA hybridization, PCR, next-generation sequencing, and quantitative 16S rRNA gene sequencing, oligonucleotide microarray based on 16S rRNA, aptly named human-microbe identification microarrays (HOMIM)	Bacterial species (*Streptococcus*, *Staphylococcus*)	[68,72,73]
Immunomics	Immunologic analysis	Immunological markers (IgM, IgA, and IgG tests, and hepatitis B virus and hepatitis C virus, IgG)	[67]

**Table 3:** Examples of single and multiplexed salivary biomarker-based PoC diagnostics [9].

PoC Platforms	System Used	Biomarker	Test Duration	Region of Origin
Single	Microelectromechanical technology (MEMS), optical fluorescent system followed by electrophoresis	Matrix metalloproteinase-8 MMP	10 min	Sandia National Lab (USA)
	Oral risk indicator ORI	MMP-8	Less than 10 min	Dentognostics (Germany)
	Chromatography test strips	HIV1&2, HCV, influenza	20 min	Orasure Technologies (USA)
	Handheld device	Cortisol, a-amylase	1 min	Nipro (Japan)
Multiplexed	Salivary diagnostics	Salivary proteins and nucleic acids	Less than 15 min	SDx (USA)
	Salivary diagnostics	IL-8 mRNA, IL-8 protein	Less than 15 min	SDx (USA)

**Table 4:** Variety of emerging PoC technologies and their clinical utility and functions.

Types of Emerging Technologies	Biomarkers/Clinical Utility	Technique Used	Function	References
Biosensors	Diabetes mellitus, (glucose biosensors), UTI, cardiac markers, acute leukaemias	Electrochemical	Drug delivery, cardio MEMS to monitor heart patients, hearing aids, insulin micropumps, endoscopic pills, retinal prosthesis	[79]
Fluorescent biosensors/FRET biosensor	Drug discovery, arthritis, cancers, cardiovascular and neurodegenerative diseases, viral infections, chronic myeloid leukaemia	Fluorescent probes are mounted through a receptor	They are able to probe gene expression, localisation of protein, signal transduction, transcription and cell cycle apoptosis	[77]
Biological Microelectromechanical Systems (BioMEMS)	Drug delivery, cardio MEMS, insulin micro pumps, endoscopic pills, retinal prosthesis	lab-on-a-chip systems/micro/nano-scale fabrication	Detection of, proteins, viruses, DNA and microorganisms	[80,81]
Microfluidics/paper based technology	Stomach cancer biomarkers (*H. pylori*), detection of urine metabolites, blood glucose, pH value, liver function, infectious agents	Optoelectronic and microfluidic system	DNA extraction, polymerase chain reaction (PCR) amplification	[83]
Electric field induced release and measurement EFIRM	IL-8 protein and IL-8 mRNA markers for oral cancer, non-squamous cell lung cancer (NSCLC) oncogenic mutation, EGFR mutation in no small cell lung cancer	Electrochemical	Liquid biopsy technique, selective hybridization	[57,84]
Smartphone based biosensors	Blood samples of falciparum malaria infected and fluorescent images *M. tuberculosis*-positive sputum smears, self-monitoring of blood glucose, cancer	Metal-oxide semiconductor (CMOS)-based photo cameras, optical-based methods including absorbance, chemiluminescence, fluorescence, reflectance, surface plasmon resonance (SPR), bio- and electrochemilumines-cence	Detector system for reflectance, colorimetry and luminescence	[85]
